# Extended-spectrum β-lactamase *Klebsiella pneumoniae* meningitis treated with tigecycline

**DOI:** 10.4103/0256-4947.51776

**Published:** 2009

**Authors:** Jamal Ahmad Wadi, Fadi Selawi

**Affiliations:** a From the Jordan Hospital & Medical Center, Amman, Jordan; b From The Specialty Hospital, Amman, Jordan

**To the Editor:** Multidrug-resistant bacteria are increasing in prevalence, causing sporadic outbreaks of difficult-to-treat infections. *Klebsiella pneumoniae* is a gram-negative pathogen that is known to cause both community and nosocomial infections. Infections caused by *Klebsiella* that express extended-spectrum β-lactamases (ESBLs) pose a serious challenge to clinicians as they are resistant to a broad range of β-lactams, including third-generation cephalosporins, thus complicating therapy and limiting treatment options. Furthermore, patients infected with these strains may have higher mortality rates and may require a longer hospital stay. Patients are generally sicker and have received more antibiotics than patients who are not infected with ESBL-producing strains.^1^,^2^

We treated a case of ESBL-positive *Klebsiella meningitis* induced by gunshot trauma, with difficult to remove shrapnel. The patient improved with 2 weeks of tigecycline treatment. Earlier we reported a case of multidrug resistant *Acinetobacter* nosocomial meningitis treated successfully with tigecycline.^3^

A 32-year-old male patient not known to have any chronic illnesses, admitted on 31 August 2008 with a history of a gunshot injury to his lumbar area with opened and shredded meninges. The large bowel was also injured; he underwent laparotomy and bowel resection with primary anastomosis with successful outcome. Many pieces of shrapnel could not be surgically removed and were left in the meninges and spinal canal. He presented with fever and leaking infected cerebrospinal fluid (CSF) from an open lumbar wound, and was taking several different antimicrobial agents (ceftriaxone, metronidazole and ciprofloxacin for a week) without good response. He suffered from bilateral lower limb paraplegia and sensory loss below the umbilical level. Several debridement surgeries and meningeal repairs were attempted, but he continued to suffer from an infected CSF leak. Surface swabs grew ESBL-positive *Klebsiella pneumonia* and *Enterococcus fecalis.* Serial CSF sampling from the upper thoracic spine showed repeated growth of *K pneumoniae* with a similar antibiogram, i.e., sensitive to imipenem, ertapenem, levofloxacin, and ciprofloxacin (tigecycline sensitivity was not available either by disk diffusion or Vitek in the specialty hospital laboratory) (Table 1). Blood cultures taken twice one week apart showed no growth. The patient was initially started on ceftriaxone, ciprofloxacine, teichoplanin, and pipracillin/tazobactam at different intervals during his admission, and then on imipenem plus teichoplanin, but the latter was discontinued as soon as ESBL-*Klebsiella* was identified from CSF. The patient had seizures that were not controlled. Six days later imipenem was discontinued and tigecycline 100 mg loading dose and 50 mg every 12 hours was prescribed for a total duration of 14 days. A week later, parenteral ciprofloxacin was added for 3 days for systemic sepsis. In the meantime he was receiving tigecycline. Two days later, the CSF culture was sterile. The patient was continued on tigecycline and showed remarkable clinical improvement. A CSF culture follow up showed progressive improvement (Table 1). He was discharged home afebrile and in relatively good condition. He was well for 2 weeks and then was readmitted with chest pain and shortness of breath, and diagnosed with pulmonary embolism to which he succumbed in 3 days.

**Table 1:** Results of cerebrospinal fluid sampling.

	8 Aug	16 Sep	20 Sep	25 Sep
Glucose (mg/dL)	11	54	47	55
Protein (mg/dL)	27	62	95	21
White blood cells (per mm^3^)	5200	340	550	90
Differential	P95%, L5%	P33%, L63%	P77%, L23%	
Red blood cells (per mm^3^)	1152	120	20	60
Culture	ESBL-producing *Klebsiella*	ESBL-producing *Klebsiella*	ESBL-producing *Klebsiella*	No growth

Shading represents dates when patient received tigecycline, which was continued for 2 additional weeks. ESBL: extended-spectrum β-lactamase. P: polymorphonuclear leukocytes, L: lymphocytes.

Tigecycline is relatively a novel glycylcycline molecule that escapes the classic resistance mechanisms that bacteria harbor against tetracyclines, i.e. drug efflux pumps and protection of ribosomes. Thus, it has expanded antimicrobial coverage and potent in vitro antibacterial activity against a wide range of clinically important gram-positive and gram-negative aerobic bacteria and anaerobes, including *Staphylococcus aureus, Enterococcus* species, *Streptococcus pneumoniae, Haemophilus influenzae, Moraxella catarrhalis, Neisseria gonorrhoeae,* most Enterobacteriaceae, and *Bacteroides fragilis,* but it has limited or no activity against *Pseudomonas aeruginosa* and reduced activity against *Proteus mirabilis.* Its licensed indications so far are skin and soft tissue infections and complicated intra-abdominal infections. Studies are currently ongoing for diabetic foot infection, community-acquired pneumonia and nosocomially acquired pneumionia (Wyeth communication). Thus far no controlled studies are available on treating CNS infections with tigecycline in humans.^3^–^6^ This is our second case of multidrug-resistant gram-negative meningitis in which tigecycline was used with a successful outcome.

Jamal Wadi serves as a consultant for Al Hikma Pharmaceuticals and receives an honorarium from Wyeth, Aventis, MSD, Al Hikma Pharmaceuticals and Abbot for lectures, meetings and consultations. He is also on an advisory board for Wyeth and Aventis.
